# Optimizing Treatment in Refractory Copper Deficiency: A Case Report

**DOI:** 10.7759/cureus.103068

**Published:** 2026-02-05

**Authors:** Bishara Jahshan, Rami B Moussa, Abdo Haddad

**Affiliations:** 1 Internal Medicine, Winchester Medical Center, Winchester, USA; 2 Internal Medicine, Lake Erie College of Osteopathic Medicine, Erie, USA; 3 Hematology and Oncology, Cleveland Clinic Fairview Hospital, Cleveland, USA

**Keywords:** anemia, copper deficiency, hypocupremia, parenteral copper therapy, roux-en y

## Abstract

Copper deficiency, though rare, is increasingly observed in patients who have undergone Roux-en-Y gastric bypass due to impaired absorption in the stomach, proximal duodenum, and small intestine. Deficiency may manifest as anemia, cytopenias, and myeloneuropathy. Standard treatment includes oral or intravenous (IV) copper supplementation, with IV replacement preferred in cases of malabsorption.

We present a case of treatment-resistant copper deficiency in a woman in her late forties with a history of Roux-en-Y gastric bypass and iron-deficiency anemia, who presented with lower extremity paresthesias. Laboratory evaluation revealed severe copper deficiency (serum copper: 10 µg/dL; normal 80-155 µg/dL). Despite oral supplementation and the standard IV copper regimen (2 mg daily for five days every four to six weeks) over a two-year period, her copper levels remained low. A revised regimen of IV copper at 2 mg twice weekly resulted in a rapid normalization of her copper levels.

This case highlights the potential limitations of standard copper replacement protocols in post-gastric bypass patients. Adjusting the frequency of IV copper administration may improve outcomes in cases of refractory copper deficiency. Individualized treatment regimens should be considered when standard protocols are ineffective.

## Introduction

Although rare, copper deficiency has increased in prevalence. This is due to an increase in gastric bypass surgeries, which impacts the absorption of many vitamins and minerals [1]. The absorption of copper occurs in the stomach, proximal duodenum, and small intestine [2]. As a result, gastric bypass surgery is one of the leading causes of copper deficiency. Having copper deficiency negatively impacts the human body and can lead to myeloneuropathy, gait disorders, anemia, and other cytopenias [3]. Intravenous (IV) supplementation is the recommended treatment when malabsorption is the suspected cause of deficiency. The dosage is typically 1-2 mg of IV copper daily for the duration of five to six days [4].

Here, we describe a case of severe copper deficiency that posed therapeutic challenges, highlighting considerations for alternative IV copper supplementation strategies.

## Case presentation

A woman in her late forties with a past medical history of hypertension, morbid obesity, obstructive sleep apnea, and systemic lupus erythematosus presented to the hematology clinic for iron-deficiency anemia.

She reported several months of paresthesias in her lower extremities without muscle weakness or ataxia. She had undergone Roux-en-Y gastric bypass five years prior and was managing iron-deficiency anemia with oral iron supplementation. She neither smoked nor used alcohol or illicit drugs.

During the physical examination, it was observed that the patient was obese. There was a slight reduction in sensation noted in her bilateral lower extremities. Her cranial nerves were intact. Her muscle strength was normal. She exhibited normal reflexes, coordination, and gait. Blood work demonstrated iron deficiency and a low copper level of 10 µg/dL (normal: 80-155 µg/dL). 

She was initially treated with IV iron sucrose and oral copper citrate supplementation, providing 2 mg of elemental copper twice daily, was administered for three months. At three-month follow-up, her iron deficiency resolved, but copper levels remained low. A more aggressive copper replacement regimen was initiated, adding IV copper (2 mg daily for 5 days every four to six weeks) in addition to oral supplementation. Despite nearly five years on this regimen, her copper levels remained persistently low (see Table 1). Zinc, B12, and iron levels were monitored and repleted as needed.

**Table 1:** Laboratory analysis Follow-up month 1 corresponds to the initial clinical visit. Subsequent follow-up months represent elapsed time since the initial visit.

Follow-up Month	Copper (µg/dL) (Reference range: 80-155 µg/dL)
1	10
4	32
9	10
20	35
26	10
42	10
46	36
48	45
50	58
52	55
55	48
57	37
59	43
60	55
61	58
65	10
67	21
68	40
70	18
71	34
72	44
74	35
75	41
76	46
78	20
79	60
80	52
81	78
82	66
85	100

**Figure 1:**
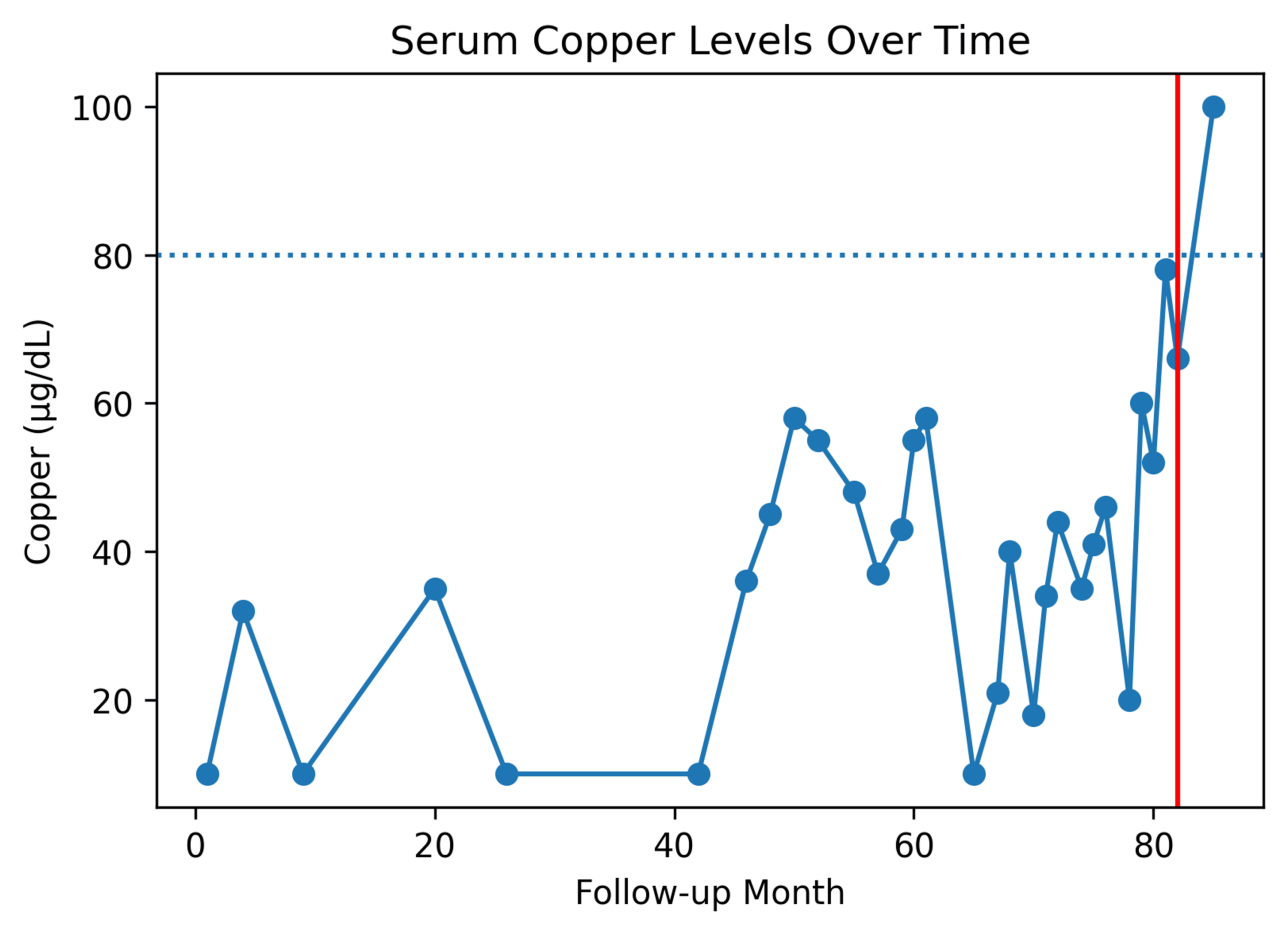
Trend of serum copper levels over time. Serum copper concentrations are shown across follow-up months. The dotted horizontal line represents the lower limit of normal copper levels (80 µg/dL). The red vertical line at follow-up month 82 indicates initiation of twice-weekly intravenous copper replacement therapy.

Eventually, her regimen was changed to 2 mg IV twice weekly for three months, resulting in normalization of copper levels (see Figure 1). After achieving normal levels, the patient was transitioned to a once-weekly IV maintenance regimen.

## Discussion

The recommended dietary allowance of elemental copper for adults is 900 μg per day [5]. Copper is abundant in nuts, organ meats, seeds, and seafood [6]. It is estimated that the incidence of copper deficiency in patients who undergo Roux-en-Y gastric bypass to reach 9.6% [7].

Standard management begins with oral supplementation. If oral therapy is ineffective, particularly with malabsorption, IV copper (2-4 mg/day for six days) is recommended [4]. Published case reports illustrate that copper deficiency often presents with cytopenias and neurologic deficits. These cases show rapid correction with copper replacement, though neurologic recovery is incomplete in some patients [8,9]. In contrast, our patient demonstrates a rare instance of persistent copper deficiency despite prolonged IV and oral supplementation over several years.

Several factors may contribute to treatment resistance, including altered absorption after bariatric surgery and interactions with other micronutrients. Excess zinc can impair copper absorption, making it a recognized cause of secondary copper deficiency [10]. Copper also plays a central role in iron metabolism, so iron and copper deficiencies often coexist [11]. In addition, copper deficiency may resemble or overlap with vitamin B12 deficiency, particularly neurologically, underscoring the need for coordinated monitoring [12,13]. In our case, zinc, iron, and vitamin B12 levels were regularly assessed and corrected throughout the patient’s course.

After years of standard replacement therapy, adjusting the intravenous copper regimen from 2 mg IV daily for five days to 2 mg IV twice-weekly dosing resulted in a significant improvement in persistent copper deficiency. This raises the possibility of more efficient copper repletion regimens in resistant cases.

## Conclusions

This case highlights the importance of screening for copper deficiency in gastric bypass patients. What makes it particularly noteworthy is its resistance to standard copper replacement protocols. These observations suggest that adjusting the frequency of IV copper administration may improve the likelihood of achieving repletion and could represent a more effective approach in refractory cases. Individualized dosing schedules should therefore be considered when conventional treatment proves inadequate. These findings may pave the way for more personalized and effective treatment protocols.
